# The potential of multi-compound nanoparticles to bypass drug resistance in cancer

**DOI:** 10.1007/s00280-017-3427-1

**Published:** 2017-09-08

**Authors:** C. G. Da Silva, Godefridus J. Peters, Ferry Ossendorp, Luis J. Cruz

**Affiliations:** 1 Translational Nanobiomaterials and Imaging, Department of Radiology, Bldg.1, C2-187h, Leiden University Medical Centre, Albinusdreef 2, 2333 ZA Leiden, The Netherlands; 2 Department of Medical Oncology, VU University Medical Center, Amsterdam, The Netherlands; 3 Department of Immunohematology and Blood Transfusion, Leiden University Medical Centre, Leiden, The Netherlands

**Keywords:** Multi-compound nanoparticles, Resistance mechanisms in cancer, Cancer, Protein kinase inhibitor, siRNA, Drug delivery, Targeting, Drug release, Nanomedicine

## Abstract

**Purpose:**

The therapeutic efficacy of conventional chemotherapy against several solid tumors is generally limited and this is often due to the development of resistance or poor delivery of the drugs to the tumor. Mechanisms of resistance may vary between cancer types. However, with current development of genetic analyses, imaging, and novel delivery systems, we may be able to characterize and bypass resistance, e.g., by inhibition of the right target at the tumor site. Therefore, combined drug treatments, where one drug will revert or obstruct the development of resistance and the other will concurrently kill the cancer cell, are rational solutions. However, drug exposure of one drug will defer greatly from the other due to their physicochemical properties. In this sense, multi-compound nanoparticles are an excellent modality to equalize drug exposure, i.e., one common physicochemical profile. In this review, we will discuss novel approaches that employ nanoparticle technology that addresses specific mechanisms of resistance in cancer.

**Methods:**

The PubMed literature was consulted and reviewed.

**Results:**

Nanoparticle technology is emerging as a dexterous solution that may address several forms of resistance in cancer. For instance, we discuss advances that address mechanisms of resistance with multi-compound nanoparticles which co-deliver chemotherapeutics with an anti-resistance agent. Promising anti-resistance agents are (1) targeted in vivo gene silencing methods aimed to disrupt key resistance gene expression or (2) protein kinase inhibitors to disrupt key resistance pathways or (3) efflux pumps inhibitors to limit drug cellular efflux.

## Introduction

Nanoparticles are emerging as ideal candidates for targeted delivery of drugs. A novel development is nanoparticles capable to encapsulate or bind multiple compounds at once and release the drugs at the target site either simultaneously or in a predetermined sequence. Nanoparticles are commonly composed of organic or inorganic materials with sizes ranging from 10 to 1000 nm (nm) and are generally 500 nm or smaller. Organic nanoparticles are usually composed of biodegradable polymers [1–5] or lipids [6], whereas inorganic nanoparticles are usually composed of gold, silver, titanium dioxide, iron, carbon, or silicon [7–9].

Nanoparticles as drug delivery agents have several advantages compared to ‘free’ drugs, including reduced bio-distribution, sustained and slow release, and protect drugs against degradation thereby prolonging drug half-life. A tissue wide bio-distribution of a drug is often unwanted, as the drug will not only go to the site of interest but also go to many other tissues, inducing dose limiting side effects. The consequence is that the critical dose is therefore not attainable and the efficacy of the drug is reduced. In contrast to ‘free’ drugs, nanoparticles can increase drug blood circulation time considerably by protecting the drug from rapid catabolism by detoxification enzymes and body clearance. In addition, nanoparticles can widen the drug repertoire to the clinic to include abandoned potent putative drugs. These include drugs with (1) a low therapeutic index, or (2) that are very hydrophobic and due to poor solubility were regarded as unsafe for in vivo application, or (3) in their ‘free’ form that would be degraded too rapidly, or (4) that become instable, or (5) that accumulate in organs of disinterest thereby inducing severe toxicity. Nanoparticles are also increasingly modified with targeting moieties to mitigate side effects to increase their efficacy even further. The targeting moieties are designed to increase cell type specificity by targeting molecules such as peptides, ligands, or antibodies to cell-specific receptors thereby enhancing specific uptake by receptor-mediated endocytosis or increasing local retention time.

Nanoparticles have clear advantages and their adoption for medical usage is emerging, as more than 40 therapeutic nanoparticles have been approved for the application in the clinic worldwide and at least 200 more are in clinical trials [10–12]. Although nanoparticles for drug delivery have several advantages over ‘free’ drugs, there are also some disadvantages, which may differ greatly from type-to-type of nanoparticle [13, 14]. For instance, while nanoparticles may help to reduce tissue wide bio-distribution, it is also this feature that is limiting its access to tissues that are located beyond blood vessels and filter organs, which limits the application of nanoparticles for some pathologies. For the specific treatment of solid tumors, however, a phenomenon entitled ‘Enhanced Permeability and Retention’ (EPR) effect, occasionally observed in human cancers, may be exploited to circumvent this obstacle [15]. The EPR effect is characterized by leaky blood vessels at the tumor site, originating from unregulated secretion of angiogenic factors, and decreased lymphatic drainage. Although the EPR effect is not always present or found very pronounced in humans, it may be induced or augmented in some specific cases, allowing nanoparticles to extravasate and still gain access to cancer cells [16–18]. An important disadvantage of some nanoparticle types is possibly organ specific toxicity, due to their propensity to accumulate in filter organs, such as liver and kidney, or spleen and lungs, although the degree of accumulation may vary considerably from type-to-type [19]. Nanoparticle surface modifications, such as amalgamation of polyethylene glycol (PEG) polymer chains (PEGylation; PEG) or adjusting the physicochemical properties, can attenuate this accumulation and therefore reduce toxicity in these organs. However, the demand for innovation maintains the pressure to continuously design novel and dexterous formulations to overcome these disadvantages and further exalt the therapeutic potential of nanoparticles to the clinic [20, 21]. For the treatment of cancer, several genotoxic and cytotoxic drugs are being encapsulated into or bound to nanoparticles to increase their efficacy and reduce side effects. For example, Doxil^®^, Abraxane^®^, and more recently Onivyde^®^ were approved and are clinically available nanoparticle formulations of doxorubicin, paclitaxel, and irinotecan, respectively. These modalities may be superior to their ‘free’ counterparts, either because of their specific delivery preventing, e.g., cardiotoxicity (Doxil) or activation at their target site. However, they do not specifically deal with existing or evolving mechanisms of resistance. As treatment resistance commonly arises in cancer, there is a dire need of a more ‘sophisticated’ class of drugs that are able to address these treatment impediments. Here onwards, this review will focus on recent developments of (multi-compound) nanoparticle modalities that, in addition to kill cancer cells, may be employed to prevent or circumvent evolving mechanisms of resistance in cancer.

## Mechanisms of resistance in cancer

A few cancer types, such as chorionic carcinoma, seminoma, and some (sub) types of lymphoma, actually respond well to cytostatic chemotherapy, commonly leading to clinical remission and cancer cures. Unfortunately, the vast majority of solid tumors will not respond as favorably. This is generally mediated by intrinsic resistance to cytotoxic drugs after an initial reduction of the tumor mass [22]. As tumors are quite heterogeneous of composition, it is not completely clear whether drug resistance is attained exclusively by clonal selection, i.e., selection of mutants resistant to the drug, a certain degree of adaptation or both [23]. Common mechanisms of resistance include pathway rewiring to accommodate enhanced proliferation, anti-apoptosis, and pro-survival signals, enhanced drug efflux and reduced influx, acquired (additional) DNA mutations, enhanced DNA repair, epithelial to mesenchymal phenotype transition, epigenetic modifications, drug inactivation, and drug target alteration, among others [24].

For example, a common aberrantly activated and pharmaceutically targeted pathway in cancer is the mitogen-activated protein kinase (MAPK) signaling pathway. This pathway provides strong survival and proliferative signals, effectively antagonizing the induction of apoptosis triggered by many oncological drugs [25]. Several signaling pathways, including the MAPK pathway, converge in the activation of the c-Myc gene that is frequently found overexpressed and mutated in a vast range of cancer types [26–29]. The Myc protein is a basic helix-loop-helix transcription factor controlling efficient proliferation of somatic and germ cells. Unfortunately, Myc has been defined to be lacking targetable active sites for drugs and therefore considered ‘undruggable’ for conventional pharmaceuticals. Another protein involved in survival and conferring drug resistance in cancer is the epidermal growth factor receptor (EGFR), often found aberrantly (over)expressed in carcinomas.

A distinct and predominant mechanism of drug resistance found in cancer cells is the overexpression of specific efflux pumps. These efflux pumps are part of the ABC superfamily of transporters and can translocate substrates (drugs) from the inside to the outside of the cell, thereby reducing intracellular drug accumulation. Currently, there are 49 human ABC transporter proteins described. From these 49 transporters, 15 are commonly associated with cancer and conferring resistance to chemotherapeutic agents. *P*-glycoprotein, BCRP, and MRP1 are among the most described efflux pumps to play a key role in multidrug resistance (MDR)-mediated resistance in cancer [30].

**Fig. 1:**
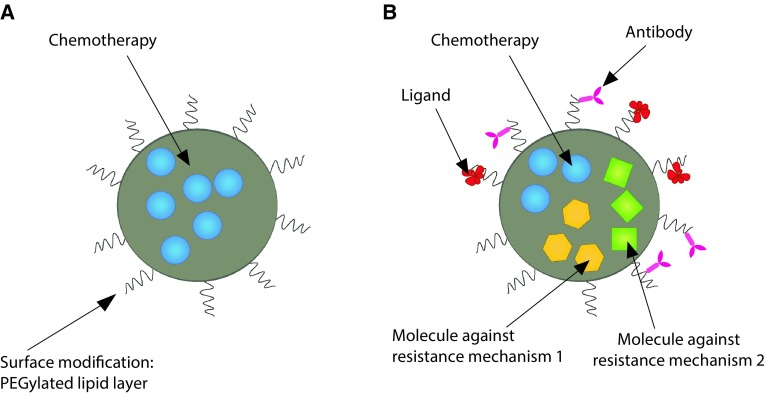
Schematic representation of distinct types of nanoparticle approaches. **a** Mono-chemotherapy nanoparticle approach containing one type of drug without targeting moieties; **b** depiction of a multidrug nanoparticle approach with active targeting moieties

Some of these mechanisms of resistance may be addressed by employing specifically adapted nanoparticles. The number of scientific publications of nanoparticles for single drug delivery is immense and their therapeutic potential is evident; however, novel strategies are required to improve cancer therapy efficacy to deal with evolving mechanisms of resistance. To achieve this goal, the delivery of several drugs, with a diverse mode of action, may be combined in nanoparticles (see Fig. 1 for an illustration). Additionally, the required level of control over the drug release time and release sequence is for this demanding task considerably higher. Notably, as the complexity of chemical assembly of such nanoparticles raises, so may the costs for GMP mass production and the costs of QA/QC [31]. In addition, the FDA/CDER approval of multi-compound nanoparticles may, in some specific cases, be more complex and slower to attain due to their polyvalent nature [31, 32].

## Targeting mechanisms of resistance with protein kinase inhibitors nanoparticle formulations

Several drugs are currently in the clinic or are being developed that can inhibit or repress specific mechanisms of resistance. These drugs are so-called small molecule inhibitors, anti-signaling drugs, or biologicals, e.g., protein kinase inhibitors (PKIs) and monoclonal antibodies. However, the cure rate of solid tumors by these modalities alone is low and acquired resistance occurs as well [33]. In addition, chronic administration is often required leading to toxicity over time. It appears that, similar to conventional chemotherapy, PKIs that are encapsulated in nanoparticles also induce less side effects, compared to ‘free’ PKI drug administration. For instance, poly (lactic-*co*-glycolic acid; PLGA) nanoparticles encapsulating erlotinib induced significantly less sub-acute toxicity in Wistar rats compared to ‘free’ administration [34]. In a similar study, Marslin et al. [35] have shown that cardiotoxicity, often a complication of prolonged administration of imatinib mesylate, could be avoided by encapsulating this drug in PLGA nanoparticles while increasing the efficacy compared to the ‘free’ drug.

**Fig. 2:**
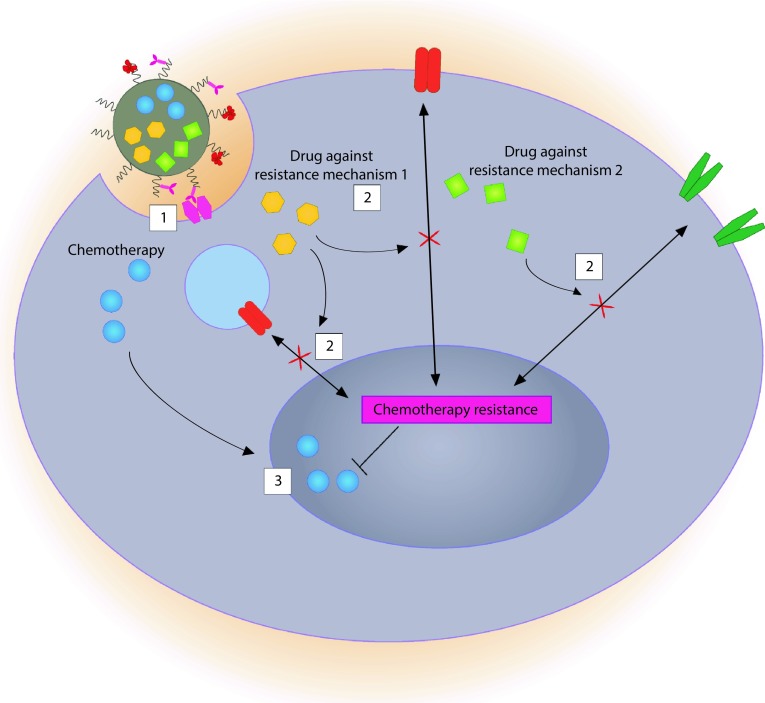
Schematic representation of a putative multi-compound nanoparticle that releases multiple compounds simultaneously or in sequence. *1* A targeted nanoparticle triggers the receptor-mediated endocytosis uptake of the nanoparticle by the target cell; *2* after intracellular processing of the nanoparticle (not depicted), selective small molecule compounds are released that inhibit mechanisms of resistance either simultaneously or in sequence, depending on the nanoparticle design. In this illustration, two distinct drugs ‘Drug against resistance mechanism’ 1 and 2 are depicted, each suppressing a different mechanism of resistance. One of these drugs could inhibit efflux pumps, to ascertain that chemotherapy is not excreted from the cell, while the second drug could suppress an (active) anti-apoptotic pathway hindering cell death related to DNA damage. *3* Cell cycle is disrupted by conventional chemotherapy by inducing DNA damage and trigger apoptosis that can now be executed unobstructed due to the inhibited anti-apoptotic pathway and the cancer cell dies

Instead of monotherapy, a rational combination of PKIs with other drugs may harbor great synergetic potential. Some combinations may enhance cancer treatment efficacy by predisposing tumors to conventional chemotherapy. For example, Basu and colleagues [36] assembled nanoparticles carrying PD98059, a selective MAPK inhibitor, to predispose cancer cells dependent on this oncogenic pathway to chemotherapy. The authors combined the nanoparticles containing PD98059 with cisplatin and observed an impressive tumor growth disparity compared to either compound alone. A clear synergistic effect was observed when these compounds were combined for simultaneous delivery to melanoma cells in vivo. Although the concurrent administration of these two modalities was beneficial in this setting, it may differ with PKI, cancer, and chemotherapy type. Lee et al. [37] have recently shown that sequential administration, but not simultaneous, may be crucial for some PKIs and conventional chemotherapy combinations. The authors showed that pre-treatment of breast cancer cells with erlotinib, a targeted EGFR inhibitor, was required to sensitize cancer cells to doxorubicin and that co-administration of both (i.e., erlotinib and doxorubicin simultaneously) was not nearly as effective. By inhibiting EGFR, the cancer cell re-acquired a working apoptosis pathway responsive to DNA damage. Furthermore, Morton et al. [38] described how liposomes could be employed to achieve such time controlled release of drugs. By loading doxorubicin into the hydrophilic core and entrapping erlotinib in the hydrophobic compartment of the membrane, erlotinib is released before doxorubicin. The sequential release effectively forces an internal rewiring of signaling pathways effected by erlotinib before DNA damage is induced by doxorubicin. This incites the cancer cell proneness toward apoptosis considerably. Figure 2 illustrates a putative modality to circumvent multiple mechanisms of resistance in cancer.

 This elegant approach achieved a much higher rate of cancer cell killing by hampering the cancer cells resistance mechanisms against apoptosis before releasing the cell-killing agent. Au et al. [39] recently showed that sequential release of drugs for cancer therapy is also possible with polymer nanoparticles, by incorporating the hydrophobic drugs wortmannin and docetaxel into an adapted formulation of PLGA-PEG nanoparticles. Wortmannin inhibits, non-exclusively, the phosphoinositide 3 kinases (PI3Ks), in essence sensitizing cancer cells to apoptosis, allowing docetaxel to successfully disrupt cell division. The PI3K and the earlier mentioned MAPK pathway are actually survival pathways preventing chemotherapeutic drugs to induce cell death; therefore, inhibition of these survival pathways will activate the chemotherapeutic drug and cell death [40]. As the molecular weight of wortmannin is lower compared to docetaxel, it was released prior to docetaxel, allowing a controlled sequential release of these drugs. Also in this setting, the pathway rewiring process before interfering with cell division was essential. Several other combined nanoparticle and protein kinase inhibitor strategies are emerging and are summarized in Table 1.

**Table 1:** Nanoparticle protein kinase inhibitor delivery targeting pathways involved in therapy resistance

Nanoparticle type	Active targeting	PKI	Primary kinase targeted	Other compounds	Model	References
Accurin polymer based	–	AZD2811	Aurora B kinase		Human colon cancer	[83]
Glutaraldehyde crosslinked albumin	Anti-EGFR nanobody^a^	17864 (platinum-bound sunitinib analog)	PDGF-R/VEGFR		Human head and neck squamous cell carcinoma (in vitro)	[84]
Gold	–	Erlotinib	EGFR		Human adenocarcinoma and non-small-cell lung cancer (in vitro)	[85]
Gold	Anti-EGFR antibody ^a^	Gefitinib	EGFR		Lung cancer (in vitro)	[86]
Hexadentate-PLGA	–	PD98059	MEK	Cisplatin (not in nanoparticle)	Melanoma and lung carcinoma	[36]
Iron oxide/magnetite	–	AM-005 + AT-9283	Aurora kinase		Liver cancer	[87]
Liposomal	–	WHI-131	JAK3/EGFR		Human B-lineage ALL/breast cancer	[88, 89]
Liposomal	−/Anti-CD19 antibody ^a^	SYK-P-site inhibitor C61	SYK		B-precursor acute lymphoblastic leukemia	[90–92]
Liposomal	Anti-EGFR nanobody ^a^	AG538	IGF-1R		Human head and neck squamous cell carcinoma and breast adenocarcinoma	[42]
Liposomal	Folate	Erlotinib	EGFR	Doxorubicin	Human breast and lung cancer	[38]
Liposomal (layer-by-layer)	CD44^a^	Selumetinib + PX-866	MEK1/2 + PI3 K		Human breast cancer	[93]
Oleic acid based	–	AZD6244	MAPK	Cisplatin	Human cervical/breast/liver cancer (in vitro)	[94]
PLGA-PEG di-block copolymer	–	Wortmannin	PI3 K	Docetaxel	Human lung and prostate cancer	[39]
PLGA	–	LY294002	PI3 K		Murine melanoma and human breast cancer	[95]
Reverse micelles	–	Erlotinib	EGFR		Pancreatic adenocarcinoma (in vitro)	[96]

*EGFR* epidermal growth factor receptor, *IGF-1R* insulin-like growth factor 1 receptor, *JAK3* Janus kinase 3, *MEK* mitogen-activated protein kinase, *PDGF-R* platelet-derived growth factor receptor, *PI3K* phosphoinositide 3-kinase, *PLGA* poly(lactic-*co*-glycolic acid), *SYK* Spleen tyrosine kinase, *VEGFR* vascular endothelial growth factor receptor

^a^Activate targeting with dual role: (1) NP targeting moiety and (2) direct perturbing mechanism of resistance by receptor agonism/antagonism or may trigger antibody mediated cytotoxicity

Cancer cells rapidly develop resistance against PKIs commonly by the activation of compensatory pathways or target site mutations [41]. For instance, it has been described that inhibition of the EGFR pathway with PKIs may eventually induce the activation of the insulin-like growth factor 1 (IGF-1R) pathway, as an acquired method of resistance. To address this adaptation, Van der Meel et al. [42] developed liposomes carrying an anti-IGF-1R kinase inhibitor and coated the liposomes with antagonistic anti-EGFR nanobodies. This approach led to considerable less pro-survival and proliferation signaling in cancer cells. The majority of the studies summarized in Table 1 describe nanoparticle approaches to target oncogenic pathways, often implicated in cancer mechanisms of resistance. Most of these studies did not combine nanoparticles with ‘conventional’ chemotherapy. However, combining PKIs with chemotherapy may hold a considerable therapeutic benefit, as indicated by the combinatorial studies described above.

In summary, several PKIs have less favorable physicochemical properties that decrease their therapeutic potential [43] and encapsulation of PKIs into nanoparticles appears to be a viable strategy to circumvent some of these limitations. In addition, PKI-associated toxicity may be reduced [34]. Nonetheless, it appears that combining nanoparticle formulated PKIs with conventional chemotherapy could be an effective strategy to hinder therapy-induced resistance. It also appears evident that the order of administration is paramount for the efficacy of the treatment modality for some cancer types; sequential rather than simultaneous and PKI exposure before cytotoxic agent.

## Targeting mechanisms of resistance with silencing RNA nanoparticle formulations

Several mechanisms of resistance in cancer have been previously targeted by suppression of specific gene expression, most commonly by small interfering RNA (siRNA) or to a lesser extent small hairpin RNA (shRNA) delivery to cancer cells. An siRNA molecule is a double-stranded RNA molecule of 20–25 base pairs whereby its sequence is complementary to a part of its target gene mRNA transcript. It is often employed to disrupt the translation of a specific gene transcript into protein by exploiting the RNA interference pathway [44].

Traditionally, gene therapy treatment with siRNA is performed by the injection of ‘naked’ siRNA directly into the bloodstream or packed in modified viruses [45, 46]. Specifically, the targeting of ‘naked’ siRNA to the cells of interest without the use of a delivery agent is generally found inefficacious as it is rapidly cleared from the bloodstream due to degradation by serum nucleases and renal clearance. In contrast, adapted viruses are, in comparison, quite efficacious delivery agents for siRNA; however, immune responses against the viral proteins abstains the efficacy of this modality [47]. As an alternative, nanoparticles can encapsulate, protect, and deliver siRNA intracellularly. Conversely, nanoparticles have limitations as well, as described in the first section of this paper, and are applicable to siRNA delivery as well, i.e., mainly the dependence of EPR effect to gain access to cancer cells in solid tumors. From an immunological perspective, immune responses against nanoparticles have been sparsely studied and may vary greatly from type and composition. For instance, nanoparticles containing 1,2-Dioleoyl-3-trimethylammonium-propane (DOTAP), mainly an ingredient for cationic liposomes, has been reported to induce potent type I and type II interferon responses [48]. However, PEGylation of nanoparticles reduces immunogenicity without the formation of any additional toxic metabolites and appears essential for successful prolonged blood circulation [20, 49]. A multifold of—mono-therapeutic—nanoparticle formulations that aim to modulate driver oncogenes have also been reported but are beyond the scope of this review. Multi-compound nanoparticles targeting specifically mechanisms of resistance by targeted siRNA gene silencing in cancer are summarized in Table 2.

**Table 2:** Nanoparticle small interfering RNA delivery targeting pathways involved in therapy resistance

Nanoparticle type	Active targeting	siRNA^a^	Compounds^a^	Model	References
Glycol chitosan	–	Bcl-2	Doxorubicin	Human prostate cancer	[50]
LCP	–	c-Myc	Gemcitabine monophosphate	Human lung cancer	[51]
LCP	Anisamide to sigma receptors	VEGF	Gemcitabine monophosphate	Human lung cancer	[52]
Liposomal	–	MRP1/BCL2	Doxorubicin	Human ovarian, breast, lung and colon cancer. (in vitro)	[97]
Liposomal	Asparagine-glycine-arginine peptide to CD13	c-Myc	Doxorubicin	Human fibrosarcoma	[98]
Liposomal	Anisamide to sigma receptors	VEGF/c-Myc	Doxorubicin	Human ovarian cancer	[99]
Liposomal	GC4 scFv antibody	c-Myc/MDM2/VEGF	miR-34a	Murine melanoma	[100]
Liposomal	–	Mcl1	SAHA (Vorinostat)	Human cervical cancer	[101]
Liposomal	–	MRP1/BCL2	Doxorubicin	Human lung cancer	[102]
Liposomal	–	BCL2	d-(KLAKLAK)_2_ peptide	Murine melanoma	[103]
Liposomal	Hyaluronic acid	MRP1	Doxorubicin	Human breast cancer	[104]
DSPE-PEG lipid	Folate	Survivin	Docetaxel	Human liver cancer	[105]
Micellar	–	HIF-1α	Doxorubicin (combined treatment; i.e., not in delivery vehicle)	Human prostate cancer	[106]
Micellar	–	Plk1	Paclitaxel	Human breast cancer	[107]
Minicell	EGFR antibody	MDR1	Doxorubicin	Uterine cancer	[108]
PDHA	–	Snail/Twist	Paclitaxel	Murine breast cancer	[109]
PEI-GO	–	Bcl-2	Doxorubicin	Human cervical cancer In vitro study only	[110]
PEO-PbAE/PCL	–	P-glycoprotein	Paclitaxel	Human ovarian cancer (in vitro)	[111]
PLGA-PEI	Biotin to biotin receptors	P-glycoprotein	Paclitaxel	Murine breast cancer	[112]
PLGA	–	DCAMKL-1	DAPT (combined treatment; i.e., not in delivery vehicle)	Human colorectal cancer	[113]
PLGA	–	REV1/REV3L	Cisplatin prodrug	Human prostate cancer	[114]
Mesoporous silica	–	Bcl-2	Doxorubicin	Human ovarian cancer (in vitro)	[115]
Mesoporous silica	–	P-glycoprotein	Doxorubicin	Human cervical cancer	[116]
Mesoporous silica	Hyaluronic acid + PEGA-pVEC peptide	CTGF	Doxorubicin	Human breast cancer	[117]

*BCL2* B cell lymphoma 2, *CTGF* connective tissue growth factor, DAPT *N*-[*N*-(3,5-difluorophenacetyl)-l-alanyl]-S-phenylglycine t-butyl ester, *DCAMKL-1* Doublecortin-like and CAM kinase-like 1, *DSPE* Distearoyl-phosphatidylethanolamine, *HIF-1α* Hypoxia-inducible factor-1α, *LCP* lipid/calcium/phosphate, *MDR1* multidrug resistance 1, *MRP1* multidrug resistance-associated protein 1, *PEI-GO* polyethylenimine-functionalized graphene oxide, *PEG* ethylene glycol, *PEO-PbAE* poly(ethylene oxide)-modified poly(beta-amino ester), *PDHA* poly[(1,4-butanediol)-diacrylate-β-5-hydroxyamylaminel, *PEO-PCL* Poly(ethylene oxide)-modified poly(epsilon-caprolactone), *PLGA* poly(lactic-*co*-glycolic acid), *PLGA-PEI* PLGA polyethyleneimine, *Plk1* polo-like kinase 1, *SAHA* suberoylanilide hydroxamic acid, *scFv* single chain fragment variable, *VEGF* vascular endothelial growth factor

^a^Compounds are encapsulated in delivery vehicles unless stated otherwise

Similar to previously discussed PKIs nanoparticle modalities, combining specific siRNA treatment with ‘conventional’ chemotherapy appears to yield superior results than any of the modalities alone. For instance, Yoon et al. [50] designed two glycol chitosan-based nanoparticles, one containing doxorubicin and another containing siRNA targeted to the Bcl-2 gene transcript. As Bcl-2 is involved in conferring resistance against apoptosis triggered by doxorubicin, durable in vivo tumor growth repression was observed after repeated injections of the nanoparticles containing doxorubicin followed by the nanoparticles containing the Bcl-2 siRNA. Moreover, combinatorial targeted nanoparticle delivery approaches of chemotherapy and siRNA targeted against (driver) oncogenes are highly anticipated. One of such oncogenes is the c-Myc gene. For instance, Zhang et al. [51, 52] recently combined gemcitabine monophosphate and c-Myc siRNA in one nanoparticle to efficiently suppress both subcutaneous and orthotopic human lung cancer growth in vivo with minimum toxicity in nude mice. As the involvement of c-Myc is quite prevalent in cancer, a prudent combinatorial nanoparticle approach of chemotherapy and c-Myc siRNA may therefore hold great potential to become one single potential treatment for a wide range of cancers of diverse etiology. The authors also combined gemcitabine monophosphate with VEGF siRNA and found increased efficacy as well. This system had an additional advantage since gemcitabine monophosphate delivery would bypass resistance due to decreased activation [53–55].

## Targeting efflux pump and other mechanisms of multidrug resistance with nanoparticle formulations

Dose escalation is a common pharmacological strategy to overcome mechanisms of resistance mediated by drug efflux pumps. While an effective approach, it is commonly accompanied by deleterious adverse effects. For instance, doxorubicin effective dose is limited by severe cardiotoxicity [56]. The upregulation of efflux pumps is a common and yet distinct method of resistance against cancer therapy. The upregulation of efflux pumps, such as the *P*-glycoprotein but more importantly MRPs and BCRP (ABCG2) [57], reduces the intracellular accumulation of specific drugs and is known to confer resistance against many chemotherapeutic agents including anthracyclines, paclitaxel, and vincristine but also several protein kinase inhibitors [58, 59]. By employing nanoparticle technology to serve as delivery agents, drug efflux is inherently reduced, as nanoparticles enter the cells mainly by endocytosis and facilitate endosomal/lysosomal escape of distinct payloads to the cytosol before their cargo is released [60–62]. Therefore, most of nanoparticle-delivered drugs are less affected by drug efflux pumps due to their location inside the cell, usually outside the reach of membrane efflux pumps [13, 63–65]. Albeit, while the drug efflux pumps are partially bypassed by encapsulating drugs in nanoparticles, the effect is not absolute, as once the drugs are released inside the cells, a portion of the drug may still become in reach of efflux pumps. In that sense, it may be prudent to actively co-inhibit efflux pumps while delivering drugs to the targets cells. For this purpose, Xu et al. [66] reported that drug efflux-mediated resistance in lung cancer cells could be effectively overcome by coating nanoparticles containing doxorubicin with cyclosporin A, which is a multimodal efflux pump inhibitor of both *P*-glycoprotein and MRP1 (ABCC1).

Alternatively, the co-delivery of a chemosensitizer, such as curcumin (diferuloylmethane), may considerably decrease drug efflux. Curcumin is a relatively non-toxic plant derived polyphenol that has been described to have anti-carcinogenic effects, mainly mediated by pathway rewiring and interfering with the cell cycle [67–70]. It is also described as a potent inhibitor of the nuclear factor kappa-light-chain-enhancer of activated B cells (NF-κB) pathway, as well as a strong suppressor of ABC transporters, including *P*-glycoprotein, MRP1, and BCRP [69, 71, 72]. However, curcumin by itself has a poor uptake by the intestinal tract and a notable low bioavailability, which makes this compound an ideal candidate to be integrated in nanoparticles approaches for targeted delivery [73]. Distinct multi-compound nanoparticle approaches that address cancer mechanisms of resistance, including curcumin co-encapsulation, are summarized in Table 3.

**Table 3:** Nanoparticle (multiple) compound delivery targeting pathways involved in therapy resistance

Nanoparticle type	Active targeting	Compounds	Primary method of resistance targeted^b^	Model	References
Amphiphilic polymer	–	Curcumin + doxorubicin	ABC pumps/NF-κB	Human multiple myeloma, acute leukemia, prostate and ovarian cancers	[74]
Cationic amphiphilic copolymer	–	IL12 plasmid + Paclitaxel	Immune suppression	Murine breast cancer	[118]
Chitosan based	–	Curcumin + doxorubicin	ABC pumps/NF-κB	Human breast cancer (in vitro)	[75]
Dendrimer	Transferrin receptor-specific peptide	TRAIL + doxorubicin	FADD	Human liver cancer	[119]
Flaxseed oil emulsion	–	Curcumin + paclitaxel	ABC pumps/NF-κB	Human ovarian adenocarcinoma (in vitro)	[120]
Gel-liposome	Hyaluronic acid	TRAIL + doxorubicin	FADD	Human breast cancer	[121]
Graphene	–	TRAIL + doxorubicin	FADD	Human lung cancer	[122]
Lipid	–	Curcumin + doxorubicin	ABC pumps/NF-κB	Human liver cancer	[123]
Liposomal	RGDK-lipopeptide	Curcumin + doxorubicin	ABC pumps/anti-angiogenic	Murine melanoma	[124]
Liposomal	DQA	Lonidamine + epirubicin (in a separate liposomal formulation)	Mitochondrial hexokinase 2	Human lung cancer	[125]
Liposomal	–	TRAIL + doxorubicin (in separate nanoparticles)	FADD	Human lung cancer	[126]
Liposomal (plus [D]-H_6_L_9_)	–	MiR-10b + paclitaxel	RhoC	Murine breast cancer	[127]
Liposomal (plus MG)	Her-2 antibody	Verapamil + doxorubicin	P-glycoprotein	Human breast cancer	[128]
Micellar based	–	Curcumin + doxorubicin	ABC pumps/NF-κB	Murine lung cancer	[129]
Micellar based	–	Disulfiram + Doxorubicin	P-glycoprotein	Human breast cancer	[130]
PCDA based	Biotin	Curcumin + doxorubicin	P-glycoprotein	Human breast cancer	[131]
PLGA based	EGFR-peptide	Paclitaxel + lonidamine	Mitochondrial hexokinase 2	Human breast and ovarian cancer	[132, 133]
PLGA	–	Cyclosporin A + doxorubicin	P-glycoprotein	Human lung cancer	[66]
PLGA	Anti-EGFR antibody^a^	Rapamycin	mTOR	Human breast cancer (in vitro)	[134]
PLGA	Folate	Nutlin-3a + curcumin	ABC pumps/NF-κB	Human retinoblastoma (in vitro)	[135]
PLGA	–	HPI-1 + Gemcitabine (Gemcitabine not in nanoparticle)	Hedgehog/Smo	Murine medulloblastoma, human pancreatic and liver cancer	[136, 137]
PLGA	–	Curcumin + doxorubicin	ABC pumps/NF-κB	Human chronic myelogenous leukemia (in vitro)	[138]
PLGA	Biotin	Tariquidar + paclitaxel	P-glycoprotein	Murine mammary tumor	[139]
PLGA	iRGD	Camptothecin + TRAIL plasmid	FADD	Human colon cancer	[140]
PLGA	Anisamide	Resveratrol + doxorubicin	ABC pumps/NF-κB	Human breast cancer	[141]

*DQA* dequalinium, *FADD* Fas-associated protein with death domain, *MG* Malachite green carbinol base, *FADD* Fas-associated protein with Death Domain, *PCDA* Poly(curcumin-dithiodipropionic acid), *PLGA* Poly(lactic-*co*-glycolic acid), *RhoC* Ras homolog gene family, member C, *TRAIL* tumor necrosis factor-related apoptosis-inducing ligand

^a^Activate targeting with dual role: (1) NP targeting moiety and (2) direct perturbing mechanism of resistance by receptor agonism/antagonism or trigger antibody induced cytotoxicity

^b^The described inhibitor mode of action is pleiotropic and may have several targets other than described

There are several studies of nanoparticle encapsulated drug combinations with curcumin available in the literature that shown efficient circumvention of multidrug resistance in a variety of models. For instance, Pramanik and colleagues [74] have shown that doxorubicin-curcumin amphiphilic polymer-based nanoparticles successfully overcome drug efflux mediated resistance, reduced cardiotoxicity, and bone marrow suppression compared to ‘free’ DOX and Doxil^®^ in several cancer models. Successful reversal of chemo sensitivity has also been described by several other groups. For example, Duan et al. [75] have reported the successful reversal of drug efflux-mediated resistance in an adriamycin-resistant cell line by the simultaneous delivery of doxorubicin and curcumin in poly (butyl cyanoacrylate) nanoparticles. The inclusion of curcumin and cytotoxic drugs in nanoparticle formulations appears to be a logical strategy to circumvent, in a non-exclusive manner, efflux pump mediated cancer therapy resistance and possibly other mechanisms of resistance, accompanied with low toxicity to non-cancerous tissue. It should, however, be mentioned that these experiments were all performed in preclinical models with relatively high induced *P*-glycoprotein expression, a condition that has not been found in patients with solid tumors, but only in some hematological malignancies.

## Conclusion and outlook

Nanoparticles are evolving from general, non-targeted, mono-drug delivery devices to become sophisticated multidrug, targeted, sequence, and time controlled drug release delivery devices. Moreover, nanoparticles can be designed to deliver drugs to cancer cells in a highly efficient manner while at the same time be able to address existing mechanisms of resistance. It is even possible to disrupt complex resistance mechanisms that require a sequence specific inhibition of pathways to bypass drug resistance. This will pave the way for the design of highly efficient, multi-functional, personalized theranostic nanomedicine [76]. This can be of immense benefit, for example, when cancer whole-genome sequencing becomes of age. This will allow specifically tailored nanoparticles to be made that can target individual cancer characteristics while therapy progression is tracked in real time by following the included imaging or reporter molecules [77]. Besides rewiring of pathways in cancer cells that overcome mechanisms of resistance to cytolytic drugs, the same design principle may be applied to modulate the tumor microenvironment. For example, modulation of specific pathways that stimulate immune suppressive cells may be interesting candidates for targeted pathway rewiring as described by Kawakami et al. [78]. The authors provide a considerable repertoire of possible targets that are involved in maintaining an immuno-suppressed environment, including STAT3, IL10, and TGFβ, or even immune modulatory antibodies [79].

As described above, several ABC efflux pumps, such as MRPs and BCRP, are upregulated in many cancer types and often found to be involved in conferring resistance against numerous oncological drugs. Several nanoparticle-based strategies have been published addressing these mechanisms of resistance (Tables 2, 3). Indeed, it appears that combining cytostatic drugs with efflux pump inhibitors increases the therapy efficacy considerably.

On the other hand, there are still obstacles that need to be overcome before nanoparticles may become successful and widely available clinical modalities [14]. Out of several, two important obstacles are: (1) the dependence of the EPR effect to gain access to target cells in solid tumors; (2) designing nanoparticles that can be assembled according to GMP regulations without becoming excessively complex and expensive to produce. These issues can be solved by emerging technologies. For instance, the dependence of the EPR effect may be effectively reduced by the design of nanoparticles that stimulate specific transcytosis [80] or combined with photodynamic therapy to enhance nanoparticle accumulation specifically in tumors [81, 82].

To conclude, when curative cancer surgery fails or is not feasible, there is currently no effective curative alternative treatment for chemotherapy resistant solid tumors. Despite the obstacles that need resolving, dexterous, and specifically formulated multi-compound and multi-functional nanoparticles may become a viable modality for the treatment of non-resectable and chemotherapy resistant cancer in the foreseeable future.
